# Wavelength-dependent Optical Instability Mechanisms and Decay Kinetics in Amorphous Oxide Thin-Film Devices

**DOI:** 10.1038/s41598-019-39744-8

**Published:** 2019-02-27

**Authors:** Junyoung Bae, Inkyung Jeong, Sungsik Lee

**Affiliations:** Department of Electronics Engineering, Pusan National University, Pusan, 46241 Republic of Korea

## Abstract

We present a study on decay kinetics for a recovery process depending on the light wavelength selected in optical instability measurements against amorphous In-Ga-Zn-O (a-IGZO) thin-film devices. To quantitatively analyze optically-induced instability behaviors, a stretched exponential function (SEF) and its inverse Laplace transform are employed for a time- and energy-dependent analysis, respectively. The analyzed results indicate that a shorter wavelength light activates electrons largely from the valence band while metastable states are deionized with the respective photon energy (*hv*). In contrast, a longer wavelength illumination is mainly activating trapped electrons at metastable states, e.g. oxygen defects. In particular, at 500 nm wavelength (*hv* ~ 2.5 eV), it shows an early persistency with a much higher activation energy. This also implies that the majority of metastable states remain ionized, thus the deionization energy >2.5 eV. However, the decay trend at 600 nm wavelength (*hv* ~ 2 eV) is found to be less persistent and lower current level compared to the case at 500 nm wavelength, suggesting the ionization energy of metastable states >2 eV. Finally, it is deduced that majority of oxygen defects before the illumination reside within the energy range between 2 eV and 2.5 eV from the conduction band edge.

## Introduction

Recently, amorphous oxide semiconductors (AOSs) have been intensively studied since they have many advantages, such as a high transparency and good uniformity^1,2^. Among AOSs, it is also known that an amorphous In-Ga-Zn-O (a-IGZO) has better electrical performances, such as a higher mobility and lower leakage current, compared to other materials^3,4^. So, the a-IGZO can be preferred for a channel layer for a higher performance thin-film transistor^5^. Despite those advantages, the AOS has issues associated with oxygen defects which influence electrical properties during illumination^6–9^. These oxygen defects are generally classified into two kinds, i.e. oxygen vacancies and oxygen interstitials^10–13^. As the first kind, oxygen vacancies are formed, where the locations of oxygens are empty in the molecular structure of AOSs, depending on the film deposition conditions, which emits two electrons under illumination. Similarly, oxygen interstitials can also exist when oxygen atoms are not participating into the molecular structure but being residing outside of it. It also emits two electrons with illumination. Interestingly, an activation energy of this optically-ionized oxygen defect becomes much higher compared to that of the non-ionized oxygen defect^13^. So, this kind of defect states with a higher activation energy is slowly recombined during a post-illumination period, thus a metastable state. Eventually, this results in the persistent photoconductivity (PPC)^13–15^. And this PPC leads to different current-decay characteristics depending on the photon energy, which remains the issue to be resolved.

In this paper, we show a theoretical investigation of optical instability mechanisms in a-IGZO thin-film devices under a visible light illumination with different wavelengths. While being based on the measured current-time characteristics for the recovery processthe stretched exponential function (SEF) and inverse Laplace transform are applied to mathematically quantify the decay behavior of the current as a function of time and energy, respectively. The analyzed results show that the current during the recovery is more persistent, and a peak of the activation energy distribution is shifted to a higher energy level when we increase the wavelength from 400 nm to 500 nm. Interestingly, however, for the case of 600 nm wavelength, the current becomes less persistent compared to the case of the 500 nm wavelength and the peak of the activation energy is even the lower. Based on these results, we finally estimate the ionization and deionization energies of metastable states are found to be around 2 eV and 2.5 eV, respectively.

## Results and Discussion

### Experimental Observation of Optical Instabilities

**Figure 1:**
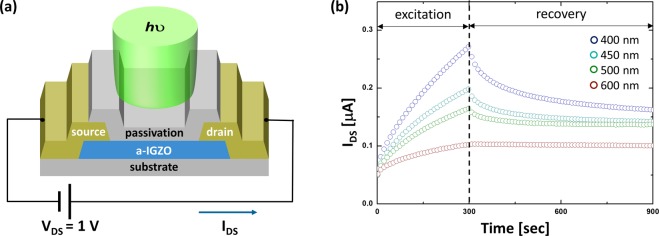
(**a**) Schematic three-dimensional view of the a-IGZO thin-film device as a two terminal resistor, examined in this work. (**b**) Measured drain current (I_DS_) vs. time (t) for different wavelengths.

Figure 1(a) shows the a-IGZO thin-film device with two electrodes fabricated with Mo (e.g. source and drain), i.e. a resistor-type. The detailed fabrication process used in this work is as follows. Note that, following a typical thin-film transistor process procedure^1–3^, starting with the glass wafer, a 50 nm-thick IGZO is formed to be used as a photo-absorption layer in the test device. Here, we used a RF-sputtering with an IGZO ceramic target. Here, we applied a low oxygen-gas partial pressure against Ar of 3%, followed by a thermal annealing at 250 °C. Here, a low oxygen-gas pressure helps to get a high electron density, thus the Ohmic conduction at the contact^1,3,16^. However, a space charge limited conduction may also happen depending on the process and illumination conditions^17,18^. Using this device, the drain-source current (I_DS_) as a function of time was measured under illuminations for different wavelengths (λ), e.g. λ = 400, 450, 500, and 600 nm, while applying the drain-source voltage (V_DS_) of 1 V, as described in Fig. 1(b). Here, an optical set-up with a light-emitting-diode is used being coupled with the KEITHLEY measuring the photo-current as a function of time, where allows a selection of 4 different wavelengths along with the same light intensity of 100 μW/cm^2^. And the peak value of the photocurrent measured at 300 sec is normalized. These optical measurements were conducted under illumination for 300 s followed by post-illumination recovery for 600 s. As the initial condition, the electron concentration (n_0_) of the IGZO film before illumination can be estimated from the initial current I_DS_(t = 0) ~ 0.05 μA, using the definitions of conductivity (*σ* ~ *qn*_0_*μ*_n_) and conductance (G = I_DS_/V_DS_ = *σ*(*S*/*L*)), yielding the following relation,

1 $${{\rm{n}}}_{0}\approx \frac{{{I}}_{{\rm{DS}}}({\rm{t}}=0\,{\rm{s}})/{{V}}_{{\rm{DS}}}}{{q}{{\mu }}_{n}({S}/{L})}.$$

here, the electron mobility *μ*_n_ is assumed to be ~20 cm^2^/V-s. With Eq. 1, n_0_ is found to be about 6.25 × 10^16^/cm^3^ for the given device geometries, such as channel length (L = 20 *μ*m) and channel cross section (*S* = 5 × 10^−6^ m^2^). Here, S is defined as Wt_s_, where W is the channel width of 100 *μ*m and t_s_ is the channel thickness of 50 nm. As seen in Fig. 1(b), during the illumination, I_DS_ is more quickly increased with a shorter wavelength light. More importantly, after the illumination, i.e. a recovery stage, their decays are relatively slow. In particular, a longer wavelength case shows a more persistent decay with a less portion of an initially-rapid decay. Comparing the current level at t = 0 s with that at t = 900 s, it is suggested that it should be related to the number of additional electrons (Δn) newly generated in the IGZO film during the illumination. For example, at 900 s, Δn for the illumination with λ = 500 nm, which is the most persistent case, is estimated based on Eq. 1 as follows,

2 $${\Delta }n\approx \frac{{{I}}_{{\rm{DS}}}({t}=900\,{\rm{s}})/{{V}}_{{\rm{DS}}}}{{q}{{\mu }}_{n}({S}/{L})}-{n}_{0}.$$

And Eq. 2 gives Δn ~ 5.38 × 10^17^/cm^3^. Here, the origin of Δn and this metastability, i.e. persistent photoconductivity (PPC), needs to be quantitatively investigated considering possible metastable states and related physical mechanisms.

### Decay Kinetics and Mathematical Analysis

**Figure 2:**
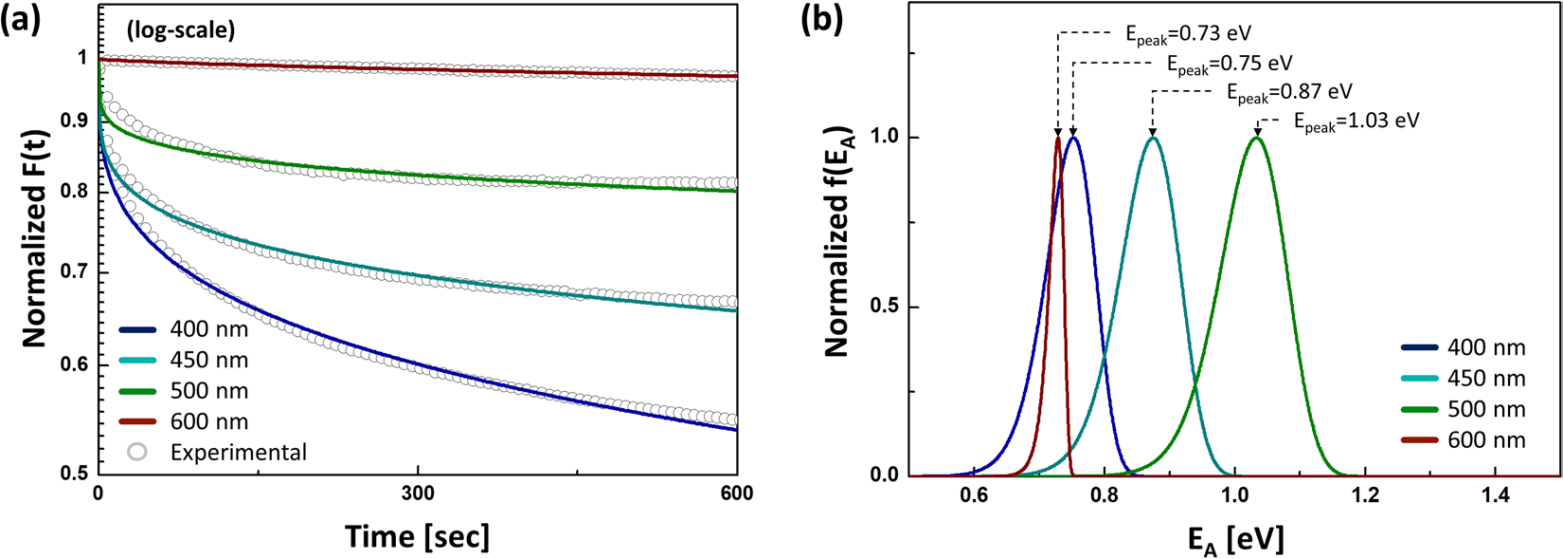
(**a**) Recovery behavior modelled with the normalized stretched exponential function with log-scale and (**b**) respective normalized activation energy distribution for different wavelength cases.

It is known that information on metastable states is associated with the recovery period where a persistency of the film conductivity appears depending on the wavelength^14,15,19^, whereas the excitation stage shows a dramatic increase regardless of wavelengths, as depicted in Fig. 1(b). Thus, in order to mathematically quantify a decay behaviour, the SEF is applied to the recovery stage, i.e. decay period (see Fig. 2(a)), rather than the excitation period, as follows^17,18^,

3 $${F}({t})=\exp (-{({t}/{{\tau }}_{{eff}})}^{{\beta }}),$$

where τ_eff_ is an effective time constant, and β is a stretched exponent. Note that an a-IGZO has many trap energy levels, so the SEF is required to analyze the decay characteristics^20,21^. It is mainly because, as seen in Fig. 2(a), the decay trend still shows non-linear characteristics in log-scale, implying that a single exponential is insufficient. And the amplitude of SEFs is normalizedfor a fair comparison across different cases. Here, the least square method is applied to extract optimum values of those parameters using MATLAB^TM^. Based on this computational procedure, parameters retrieved here are summarized in Table 1.

**Table 1:** Parameters retrieved with SEFs for respective wavelength cases during the post-illumination (i.e. recovery).

λ [nm]	400	450	500	600
β [unitless]	0.282	0.219	0.187	0.717
τ_eff_ [sec]	3.30 × 10^3^	3.17 × 10^4^	1.96 × 10^6^	8.49 × 10^4^

Based on those extracted parameters of the SEF for each case, the activation energy distribution (AED) of metastable states can also be mathematically estimated. For this, firstly, the SEF is converted to a function of frequency (f(S)) using the inverse Laplace Transform, i.e. L^−1^{*F*(*t*)}, as follows^22^,

4 $${f}({S})=\frac{1}{2\pi {j}}{\int }_{-{j}\infty }^{{j}\infty }\exp (-{(t/{\tau }_{{eff}})}^{{\beta }})\exp ({St}){dt},$$

where S is a frequency. Secondly, S in Eq. 4 can be replaced with an activation energy (E_A_) using the following Arrhenius relation,

5 $${S}={{\nu }}_{{AE}}\exp (-{{E}}_{{A}}/{kT}).$$

Here, *ν*_AE_ is an attempt-to-escape frequency and kT is the thermal energy. And the solution of Eq. 4 can be represented as a function of E_A_ while replacing S with Eq. 5, yielding,

6 $$\begin{array}{rcl}{f}({{E}}_{{A}}) & \approx  & \frac{{{\tau }}_{{eff}}{{\beta }}^{1+{\gamma }/2}}{\sqrt{2{\rm{\pi }}{\beta }({1}-{\beta })}{({{\tau }}_{{eff}}{{\nu }}_{{AE}}\exp (-{{E}}_{{A}}/{kT}))}^{1+{\gamma }/2}}\\  &  & \times \,\exp (-({1}-{\beta }){{\beta }}^{{\gamma }}/{({{\tau }}_{{eff}}{{\nu }}_{{AE}}\exp (-{{E}}_{{A}}/{kT}))}^{{\gamma }}),\end{array}$$

where γ is defined as β/(1 − β). Along with values of β and τ_eff_ seen in Table 1, we can now find the AEDs for respective cases, as shown in Fig. 2(b). Here, *ν*_AE_ is assumed to be 10^7^/sec for the a-IGZO^23^.

As shown in Fig. 2(b), it is found that a peak of the AED is usually shifted to a higher energy with increasing the wavelengthwithin the range from 400 nm to 500 nm. When we increase the wavelength further, however, the 600 nm-wavelength-light shows a narrow distribution with a peak at a lower energy in comparison even with the case of λ = 400 nm. It means that dominant metastable states are activated by illuminations at the wavelength range of 400 nm ~ 500 nm.

### Instability Mechanisms and Capture Cross-sections

**Figure 3:**
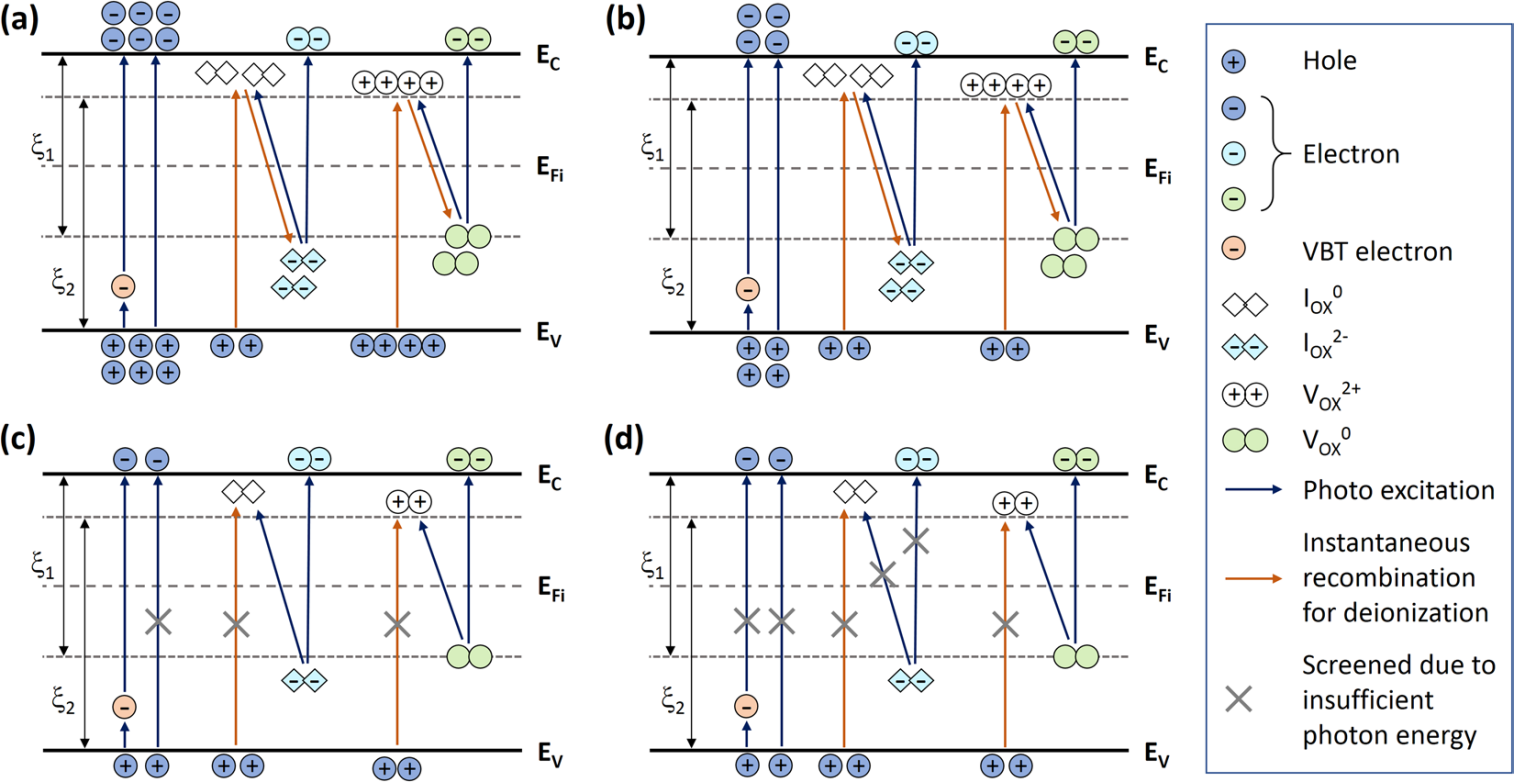
Schematic diagram of capture cross-sections for four different cases subject to four different wavelengths: (**a**) λ = 400 nm (h*ν* ~ 3.1 eV) (**b**) λ = 450 nm (h*ν* ~ 2.8 eV) (**c**) λ = 500 nm (hv ~ 2.5 eV) (**d**) λ = 600 nm (hv ~ 2.0 eV) Here, E_V_, E_Fi_, and E_C_ denote the valence band maxima, intrinsic Fermi level, and conduction band minima, respectively. And ξ_1_ and ξ_2_ are defined as the energies needed to ionize oxygen defects and to get an instantaneous recombination (i.e. deionization), respectively.

To specify a physical mechanism for respective case (see Fig. 2(b)), a capture cross-section is drawn, as seen in Fig. 3. There are two main physical mechanisms. The basic process is the band-to-band excitation, i.e. electron emission directly from the valence band (VB), and another process is associated with metastable states, such as oxygen vacancies (V_OX_°) and oxygen interstitials (I_OX_^2−^). In particular, those oxygen defects are ionized under the illumination having respective chemical reactions, i.e. V_OX_° → V_OX_^2+^  + 2e^−^ and I_OX_^2−^ → I_OX_^0^ + 2e^−^. And this gives rise to a persistent photoconductivity (PPC), i.e. metastability^15,24^. The detailed explanation for respective cases is as follow.

As shown in Fig. 3(a) for λ = 400 nm, electrons are generated from the VB as well as oxygen defect states. The photon energy (*hv*) of this case is comparable with band-gap energy (E_g_) of the a-IGZO and much bigger than ξ_1_ (i.e. ionization energy). In addition, *hv* at λ = 400 nm (~3.1 eV) is much greater than ξ_2_ (i.e. deionization energy), so that an instantaneous recombination of ionized metastable states can slightly happen with electrons supplied from the VB. As described in Fig. 3(b), the overall process at λ = 450 nm is similar to the case of λ = 400 nm. In this case, *hv* at λ = 450 nm is ~2.8 eV, which is also higher than both ξ_1_ and ξ_2_. This suggests that the ionization of metastable states as well as instantaneous recombination (i.e. deionization) for some of ionized states can simultaneously happen as well. However, its photon energy is insufficient for the band-to-band excitation. Instead, the excitation process of VB electrons may get an assistance through valence band tail (VBT) states, thus an indirect excitation (see Fig. 3(b)). Similarly, for λ = 500 nm (i.e. *hv* ~ 2.5 eV), oxygen defects can still be ionized with *hv* bigger than ξ_1_, but an instantaneous recovery process is largely screened if *hv* < ξ_2_, as shown in Fig. 3(c). In this case, likewise, the excitation of VB electrons may also slightly happen with the assistance of VBT states. This implies that majority of ionized oxygen defects cannot be deionized, corresponding to the highest E_A_, as seen in Fig. 2(b). In other words, it becomes difficult for slow metastable states to be recombined instantaneously under that illumination condition where the wavelength is longer than 500 nm. Indeed, for the case of λ = 600 nm (see Figs 2(b) and 3(d)), since *hv*(~2.0 eV) is insufficient to overcome ξ_1_, so I_OX_^2−^ and majority of V_OX_^0^ are hard to be ionized, but a small portion of the V_OX_^0^ can be ionized. And this is consistent witha smaller increase of the measured current during the illumination with λ = 600 nm (see Fig. 1(b)). Therefore, the AED for λ = 600 nm shows a narrower distribution with a peak point which is lower than other cases.

**Figure 4:**
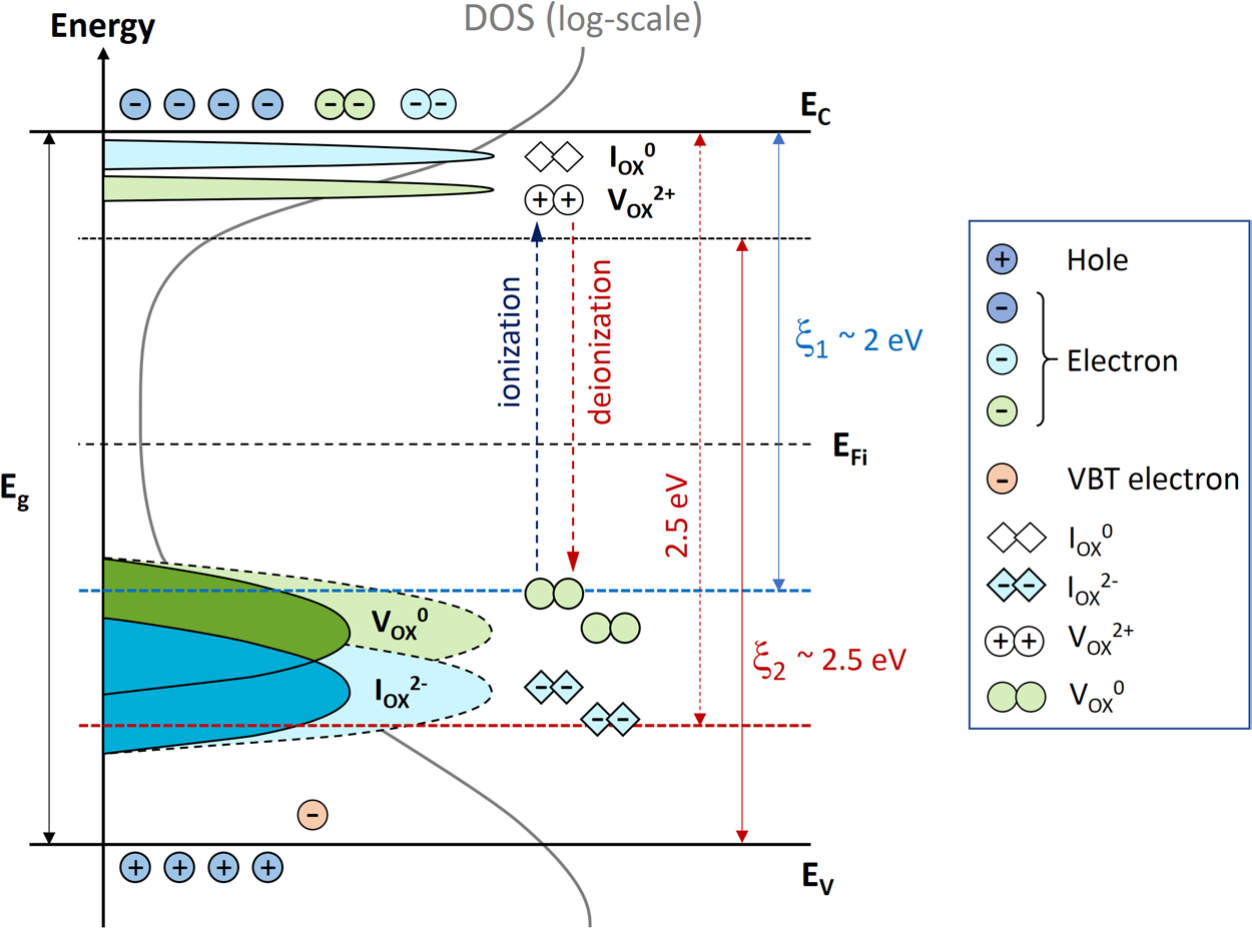
Illustration to summarize capture cross-sections related to wavelength-dependent metastability discussed with Fig. 3 (Inset: the symbols of types of defects and excitation processes).

Figure 4 shows a summary of the capture cross-section discussed with Fig. 3. This finally suggests that the ionization of metastable states dominantly happens when *hv* > ξ_1_, and their deionization (i.e. recombination) is strongly screened when *hν* < ξ_2_. So, it is implied that the case with λ = 500 nm is satisfied with both conditions, so more persistent than any others in our experiments. From these results, the ranges of the ionization and deionization energies (ξ_1_ and ξ_2_) are estimated as follows, respectively,

7 $$2.5\,{\rm{eV}}(\lambda =500\,{\rm{nm}}) > {{\rm{\xi }}}_{1} > 2.0\,{\rm{eV}}({\rm{\lambda }}=600\,{\rm{nm}}),$$

8 $$2.8\,{\rm{eV}}({\rm{\lambda }}=450\,{\rm{nm}}) > {{\rm{\xi }}}_{2} > 2.5\,{\rm{eV}}({\rm{\lambda }}=500\,{\rm{nm}}).$$

Based on Eqs 7 and 8, it is also suggested that the majority of oxygen defects before illumination reside within the energy range between −2 eV and −2.5 eV from the conduction band edge, which are consistent with the earlier reports^1,7,9,13,21,23,25^.

## Conclusions

A quantitative analysis based on a stretched exponential function and its inverse Laplace transform is presented on optical instability mechanisms and respective decay kinetics in amorphous oxide thin-film devices. The presented analysis shows that decay behaviour is largely dominated by ionized metastable states which reside near the conduction band minima. Here, their instantaneous deionization with the valence band electrons are found to be more screened as increasing the wavelength up to 500 nm (*hv* ~ 2.5 eV). This results in a more persistency as a function of time, which explained with an insufficient photon energy to reach them located near the conduction band minima, suggesting the deionization energy >2.5 eV. With the 600 nm wavelength light (*hv* ~ 2.0 eV), however, the decay trend becomes slightly less persistent compared to the 500 nm case, suggesting the ionization energy of metastable states >2 eV. These results indicate that the majority metastable states before illumination are located in the energy range between 2 eV and 2.5 eV from the conduction band edge.

## References

[1] Nomura K (2004). Room-temperature fabrication of transparent flexible thin-film transistors using amorphous oxide semiconductors. Nature.

[2] Nathan A (2012). Flexible electronics: the next ubiquitous platform. Proceedings of the IEEE.

[3] Nomura K (2006). Amorphous oxide semiconductors for high-performance flexible thin-film transistors. Jpn. J. Appl. Phys..

[4] Yabuta H (2006). High-mobility thin-film transistor with amorphous InGaZnO_4_ channel fabricated by room temperature rf-magnetron sputtering. Appl. Phys. Lett..

[5] Wager, J. F., Keszler, D. A. & Presley, R. E. *Transparent Electronics 1st edn* (Springer, 2008).

[6] Jeon S (2012). Gated three-terminal device architecture to eliminate persistent photoconductivity in oxide semiconductor photosensor arrays. Nature Materials.

[7] Nathan A (2013). Transparent oxide semiconductors for advanced display applications. Inf. Display.

[8] Nathan A (2014). Amorphous oxide semiconductor TFTs for displays and imaging. J. Disp. Tech..

[9] Lee S (2015). Transparent semiconducting oxide technology for touch free interactive flexible displays. Proceedings of the IEEE.

[10] Noh H (2011). Electronic structure of oxygen-vacancy defects in amorphous In-Ga-Zn-O semiconductors. Phys. Rev. B.

[11] Ryu B (2010). O-vacancy as the origin of negative bias illumination stress instability in amorphous In-Ga-Zn-O thin film transistors. Appl. Phys. Lett..

[12] Han WH (2015). Electronic structure of oxygen interstitial defects in amorphous In-Ga-Zn-O semiconductors and implications for device behavior. Phys. Rev. Applied.

[13] Lee S (2015). Oxygen defect-induced metastability in oxide semiconductors probed by gate pulse spectroscopy. Sci. rep..

[14] Janotti A, Van de Walle CG (2005). Oxygen vacancies in ZnO. Appl. Phys. Lett..

[15] Lany S, Zunger A (2005). Anion vacancies as a source of persistent photoconductivit**y** in II-VI and chalcopyrite semiconductors. Phys. Rev. B.

[16] Zhou HT (2015). Realization of a fast-response flexible ultraviolet photodetector employing a metal–semiconductor–metal structure InGaZnO photodiode. RSC Adv..

[17] Mihailetchi VD, Wildeman J, Blom PWM (2005). Space-Charge Limited Photocurrent. Phys. Rev. Lett..

[18] Goodman AM, Rose A (1971). Double Extraction of Uniformly Generated Electron-Hole Pairs from Insulators with Noninjecting Contacts. J. Appl. Phys..

[19] Shimakawa K, Kolobov A, Elliott SR (1995). Photo induced effects and metastability in amorphous semiconductors and insulators. Adv. Phys..

[20] Fomani AA, Nathan A (2011). Metastability mechanisms in thin film transistors quantitatively resolved using post-stress relaxation of threshold voltage. J. Appl. Phys..

[21] Luo J (2013). Transient photoresponse in amorphous In-Ga-Zn-O thin films under stretched exponential analysis. J. Appl. Phys..

[22] Wager JF (2014). An amorphous oxide semiconductor thin-film transistor route to oxide electronics. Curr. Opin. Solid State Mater. Sci..

[23] Flewitt AJ, Powell MJ (2014). A thermalization energy analysis of the threshold voltage shift in amorphous indium gallium zinc oxide thin film transistors under simultaneous negative gate bias and illumination. J. Appl. Phys..

[24] Chowdhury MDH, Migliorato P, Jang J (2010). Light induced instabilities in amorphous indium–gallium–zinc–oxide thin-film transistors. Appl. Phys. Lett..

[25] Kamiya Y, Nomura K, Hosono H (2010). Present status of amorphous In–Ga–Zn–O thin-film transistors. Sci. Technol. Adv. Mater..

